# The interplay between metabolic health factors and stroke incidence in aging populations

**DOI:** 10.3389/fendo.2025.1646643

**Published:** 2025-09-03

**Authors:** Chonghui Zhang, Tao Xiong, Kaili Ren, Hongyu Wu, Shanshan Cai, Liqin Wang

**Affiliations:** 1 Department of Blood Transfusion, The Affiliated Hospital of Qingdao University, Qingdao, China; 2 Nanjing Medical University, Nanjing, China; 3 Division of Biomedical and Life Sciences, Faculty of Health and Medicine, Lancaster University, Lancaster, United Kingdom

**Keywords:** metabolic disorders, stroke risk, diabetes, hypertension, obesity

## Abstract

**Background:**

Stroke remains a leading cause of morbidity and mortality in aging populations, and its risk is closely linked to metabolic disorders such as diabetes, hypertension, hyperlipidemia, and obesity. To better understand these relationships, this study aimed to quantify the associations between key metabolic health factors and both stroke incidence and cognitive outcomes in older adults, and to evaluate the predictive value of these metabolic factors for stroke risk through cross-sectional secondary analyses of two population-based cohort datasets.

**Methods:**

We analyzed data from the Health and Retirement Study (HRS; n = 7,322) and the English Longitudinal Study of Ageing (ELSA; n = 6,630). Associations with stroke incidence were assessed using multivariable logistic regression, and associations with cognitive outcomes were examined using multivariable linear regression. Random forest models evaluated the relative importance of metabolic factors for stroke prediction, with discrimination quantified by ROC curves (AUC). Mediation analyses explored whether stroke mediated the link between metabolic disorders and cognition.

**Results:**

Across both cohorts, diabetes and hypertension were consistently associated with higher odds of stroke, while obesity and hyperlipidemia showed smaller and cohort-dependent associations. Random forest analyses identified hypertension and diabetes as the strongest predictors of stroke. ROC analyses showed moderate discriminative performance for single metabolic factors (AUCs generally 0.70 – 0.80), with obesity performing weakest. Metabolic disorders—particularly diabetes and hypertension—were associated with worse cognitive performance, whereas hyperlipidemia showed small positive associations with certain cognitive measures in some models. Mediation analyses suggested that part of the adverse impact of metabolic disorders on cognition operates through stroke.

**Conclusions:**

Metabolic health is strongly linked to stroke risk and cognitive outcomes in older adults. Targeted detection and management of hypertension and diabetes should be prioritized to reduce stroke burden and cognitive decline. Given only moderate predictive performance of single metabolic markers, comprehensive risk models integrating socioeconomic, clinical, and lifestyle factors are warranted for improved stroke risk stratification.

## Introduction

Stroke is one of the leading causes of morbidity and mortality worldwide, posing substantial healthcare challenges and economic burdens (1). It is characterized by the abrupt loss of neurological function due to vascular disruption in the brain, which can be categorized into ischemic and hemorrhagic types (2). The pathophysiology of stroke is complex and multifactorial, encompassing various risk factors, including hypertension, diabetes mellitus, dyslipidemia, and obesity (3, 4). Among these, metabolic syndrome has gained prominence as a cluster of conditions that significantly elevate the risk of cardiovascular diseases, including stroke (5). Metabolic syndrome is defined by the presence of obesity, insulin resistance, hypertension, and dyslipidemia, collectively contributing to an increased likelihood of vascular complications (6, 7). Epidemiological studies have consistently shown a strong association between metabolic syndrome and stroke, underscoring the need for a deeper understanding of how these metabolic factors interact and contribute to cerebrovascular events (8).

Recent research has highlighted the intricate relationship between metabolic health and stroke outcomes (9). Individuals with metabolic syndrome are more likely to experience stroke due to the combined effects of elevated blood pressure, increased blood glucose levels, and lipid abnormalities (10). The risk factors associated with metabolic syndrome can lead to endothelial dysfunction, atherosclerosis, and ultimately, cerebrovascular disease (11). Furthermore, the inflammatory processes and oxidative stress induced by these metabolic abnormalities may exacerbate neuronal damage during a stroke, leading to poorer clinical outcomes and long-term disability (12). Given the escalating prevalence of obesity and associated metabolic disorders globally, particularly in aging populations, it is imperative to explore the implications of metabolic health on stroke risk and recovery (13).

Several studies have provided insights into the direct effects of individual components of metabolic syndrome on stroke risk (14). For instance, prospective cohort studies have demonstrated that diabetes is a significant risk factor for both ischemic and hemorrhagic strokes, with individuals suffering from diabetes exhibiting two to three times higher risk compared to non-diabetic counterparts (15). Hypertension, often coexisting with metabolic syndrome, is another critical risk factor, as it can lead to increased vascular resistance and subsequent stroke through its effects on vascular integrity and blood flow dynamics (16, 17). However, the interplay between these factors, particularly how they cumulatively influence cognitive and functional outcomes post-stroke, remains less explored.

Moreover, despite extensive research into the individual effects of metabolic syndrome components on stroke incidence (18), there remains a relative scarcity of studies investigating the combined effect of these factors on associated cognitive decline and overall mental health. Cognitive impairment is frequently observed following a stroke, and those with pre-existing metabolic disorders may face heightened risks of severe cognitive deficits and poorer rehabilitation outcomes (19). This underscores the need for comprehensive analyses that consider not only the direct impact of metabolic syndrome on stroke incidence but also its secondary effects on cognitive function post-stroke.

Our study aims to fill this literature gap by examining the impact of metabolic health indicators on cognitive outcomes in stroke patients. We hypothesize that metabolic syndrome’s core components significantly worsen post-stroke cognitive decline. We will explore the collective contributions of diabetes, hypertension, dyslipidemia, and obesity to cognitive impairment in stroke survivors, utilizing data from large longitudinal studies, such as the Health and Retirement Study (HRS) and the English Longitudinal Study of Ageing (ELSA).

The primary objectives are threefold: to assess the prevalence of metabolic syndrome among stroke survivors, evaluate relationships between individual metabolic risk factors and cognitive outcomes, and determine the predictive value of a comprehensive metabolic health profile for cognitive decline in stroke patients. Our findings will provide essential insights into how metabolic health interacts with cerebrovascular health and cognitive function, enhancing clinical strategies for prevention, intervention, and rehabilitation in stroke care.

In conclusion, understanding the interactions between metabolic syndrome and stroke outcomes is vital for informing public health strategies and therapeutic interventions aimed at reducing the stroke burden.

## Methodology

### Study design

This study employed a cross-sectional design aimed at exploring the relationship between metabolic health factors and stroke incidence through two complementary analytical approaches. Data was sourced from two large-scale studies: the HRS and the ELSA, which provided comprehensive demographic characteristics and health information for effective analysis. Multivariable logistic regression models were utilized for explanatory purposes to elucidate associations between individual metabolic factors (diabetes, hypertension, hyperlipidemia, and obesity) and stroke risk through odds ratios and confidence intervals. Random forest models served predictive purposes to develop stroke risk prediction tools and assess the relative importance of metabolic factors using Mean Decrease Gini indices. This dual analytical strategy enabled both understanding of causal relationships between metabolic factors and stroke (explanatory analysis) and development of clinically useful risk prediction tools (predictive analysis), providing comprehensive insights that bridge epidemiological understanding with clinical decision-making support.

### Data sources and participants

#### HRS

HRS is a long-term study established by the University of Michigan, designed to follow the health, economic status, and lifestyle of older adults (aged 50 and above) in the United States. The study began in 1992, collecting data through biannual surveys. For this analysis, we utilized the most recent round of data (2020), encompassing 7,322 participants, of whom 809 were diagnosed with stroke. Participant information included age, gender, years of education, and health status (such as diabetes, hypertension, hyperlipidemia, and obesity).

#### ELSA

ELSA is a national longitudinal study targeting adults aged 50 and over in England, with the first data collection taking place in 2002. The study aims to investigate the health, economic, and social conditions of older adults. Compared to HRS, ELSA places more emphasis on mental health and social welfare impacts, particularly concerning cognitive ability assessments. We examined data from 6,630 participants, among whom 369 were recorded as stroke patients, and 6,291 participants served as controls, providing robust support for comparative analysis.

#### Variable definition and measurement

Key variables analyzed in this study included:

Stroke: Determined based on participants’ medical history and self-reporting. Individuals recorded as having had a stroke were classified as the stroke group, while others were classified as controls. The definition of stroke included both ischemic and hemorrhagic strokes.

Metabolic health indicators:

1. abetes: Self-reported history of diabetes, classified according to World Health Organization (WHO) criteria. Participants were classified as diabetic based on self-reported physician diagnosis of diabetes, current use of diabetes medications (insulin or oral hypoglycemic agents), or fasting plasma glucose ≥126 mg/dL (7.0 mmol/L). Coded as present (1) or absent (0).

2. pertension: Determined by self-report or medication usage, defined using American Heart Association (AHA) guidelines. Participants were classified as hypertensive based on self-reported physician diagnosis, current use of antihypertensive medications, or measured blood pressure ≥140/90 mmHg. Coded as hypertensive (1) or normotensive (0).

3. perlipidemia: Classified according to National Cholesterol Education Program Adult Treatment Panel III (NCEP ATP III) guidelines. Determined as present based on blood tests showing total cholesterol ≥240 mg/dL (6.2 mmol/L), self-reported physician diagnosis, or current use of lipid-lowering medications. Coded as present (1) or absent (0).

4. esity: Defined based on Body Mass Index (BMI) calculated from participants’ self-reported weight and height, using World Health Organization (WHO) criteria. BMI ≥30 kg/m² was categorized as obese. Coded as present (1) or absent (0).

Cognitive assessment tools:

Cognitive scores were measured through standardized tests assessing participants’ cognitive abilities. The study utilized the following four scoring measures:

1. gnitive Decline: Scoring based on cognitive function assessments including memory and attention tasks, measured using standardized cognitive batteries with immediate and delayed word recall tests.

2. ntal Status: Score measuring the level of psychological health, including symptoms of anxiety and depression, assessed using the Mini-Mental State Examination (MMSE) for HRS participants and comparable cognitive screening questions adapted for ELSA participants.

3. eech Function: Scoring assessing fluency and communication ability, evaluated through verbal fluency tests measuring phonemic and semantic fluency according to standardized neuropsychological assessment protocols.

4. gnitive Score: An aggregate score reflecting overall cognitive competence across all assessments, derived from standardized cognitive test batteries and normalized to population means, with higher scores indicating better cognitive performance.

Socioeconomic variables:

1. et Family Wealth: The total assets of a family after liabilities, indicating financial stability. Measured in US dollars and used as a continuous variable in analyses.

2. tal Family Income: Combined income of all household members, reflecting economic capability and access to healthcare. Measured in US dollars annually and included as a covariate to control for socioeconomic status.

### Cognitive assessment methods

HRS Cognitive Measures: Cognitive function in HRS was assessed using standardized protocols based on the Langa-Weir classification system. The Total Cognitive Ability Score (range: 0 – 35 or 0 – 27 points) was calculated by combining immediate word recall (10 common words), delayed word recall (after 10 – 15 minutes), mental status assessment (date orientation, object naming), and backwards counting (from 20 to 10). The Total Mental Status Score (range: 0 – 10 points) evaluated executive function and orientation through temporal orientation (current date, month, year, day of week), spatial orientation (current state, county), object naming (common items such as scissors, cactus), and presidential naming (current and previous U.S. presidents). The Cognitive Ability Score provided a comprehensive assessment of memory, attention, and executive function through working memory tasks, attention concentration tests, verbal fluency tests, and abstract thinking abilities. All scores were standardized with higher scores indicating better cognitive performance.

ELSA Cognitive Measures: ELSA employed longitudinal cognitive assessments to track cognitive changes over time. Cognitive Decline was calculated through longitudinal comparisons using standardized Z-scores, incorporating word list learning and recall, executive function tests (attention switching, working memory), and language abilities (vocabulary tests, verbal fluency), with negative values indicating cognitive deterioration. Mental Status (range: 0 – 10 points) was assessed using Mini-Mental State Examination-related items including temporal and spatial orientation, attention and calculation abilities, and language comprehension and expression. Speech Function was evaluated through phonemic fluency (words beginning with specific letters within time limits), semantic fluency (animal naming test), and language comprehension (instruction execution ability), with scores reflecting the number of correct responses. The overall Cognitive Score represented a standardized composite measure combining memory function, executive function, attention, and language function scores, standardized through Z-scores to facilitate longitudinal comparisons. All ELSA cognitive measures underwent age and education adjustments using population norms.

### Data processing and variable preparation

Data Cleaning and Quality Control: Prior to analysis, comprehensive data cleaning procedures were implemented for both HRS and ELSA datasets. Participants with incomplete demographic information (age, sex, education) were excluded from the analysis. For cognitive outcome variables, participants missing more than 50% of cognitive test components were excluded to ensure data integrity. Outliers were identified using the interquartile range (IQR) method, with values exceeding 3×IQR beyond the 25th or 75th percentiles flagged for review and verified against original data sources. Variables were checked for logical consistency (e.g., diagnosis dates preceding study enrollment), and implausible values were cross-validated with medical records where available.

Missing Data Management: Missing data patterns were systematically evaluated using Little’s MCAR test to assess randomness of missingness. For metabolic disorder variables (diabetes, hypertension, hyperlipidemia, obesity), missing values were handled through multiple imputation using chained equations (MICE) with 20 imputations, incorporating demographic variables, baseline health status, and available clinical measurements as predictors. Cognitive outcome variables with sporadic missing values (<10% missingness) were imputed using the same multiple imputation framework, while cases with extensive cognitive data missingness (>50%) were excluded from the analysis. Sensitivity analyses were conducted comparing complete case analysis with multiple imputation results to assess the robustness of findings. For longitudinal cognitive assessments in ELSA, linear mixed-effects models were employed to handle missing follow-up data under the missing at random (MAR) assumption, utilizing all available time points for each participant to maximize statistical power and minimize bias.

### Data analysis methods

Data analysis was performed using R programming language (version 4.0.0), with the following analytical procedures:

#### Descriptive statistics

Initial descriptive statistical analyses were conducted for all variables, including participants’ demographic characteristics (such as age, gender, education), health status, and cognitive scores. Descriptive statistics provide basic insights into the sample’s characteristics and help understand data distribution.

### Outlier and missing value handling

Before analysis, outliers and missing values within the data were examined. Boxplots and scatterplots were utilized to visualize and identify outliers, and decisions were made based on specific cases regarding whether to correct, delete, or retain them. For a small number of missing values, imputation methods (mean or median imputation) were used, while multiple imputation methods were considered when the missing value proportion was high.

### Multivariable linear regression model construction

We employed a multivariable linear regression model to investigate the impact of metabolic factors on cognitive scores.

We analyzed the impact of different metabolic health factors on cognitive ability through this model. Additionally, we utilized stepwise regression (Backward Elimination) based on significance to identify relevant variables and build a simplified model.

### Random Forest analysis

In addition to multivariable linear regression, we utilized Random Forest (RF) as a complementary analytical tool. Random Forest is an ensemble learning method based on decision trees, adept at handling large datasets and classification problems. It enhances model accuracy and stability by building multiple decision trees and aggregating their predictions. In this study, Random Forest was used to assess the importance of metabolic health factors on four cognitive scores (Cognitive Decline, Mental Status, Speech Function, and Cognitive Score).

### Random Forest model construction

1. Feature Selection: Key metabolic indicators (diabetes, hypertension, hyperlipidemia, and obesity) were selected as independent variables, with cognitive scores serving as the dependent variable for constructing multiple decision trees.
2. Model Training: Utilizing the randomForest package in R for model training with a pre-set number of trees (ntree) at 500 to ensure model stability.
3. Feature Importance Evaluation: After model training, the contribution of each feature to the model’s predictions (using the Gini index) was computed to identify the metabolic health factors most impacting cognitive ability.

### Model evaluation

After completing both regression and random forest analyses, we evaluated the models’ fit. Key metrics included:

1. R² (Coefficient of Determination): Indicates the proportion of variance explained by the model; higher values indicate a better fit.
2. Adjusted R²: Accounts for the number of variables included in the model, suitable for comparing models with different numbers of features.
3. F-statistic: Tests the overall significance of the model to evaluate the collective impact of the independent variables on the dependent variable.

For assessing the significance of regression coefficients, we adopted a significance level (generally set at 0.05), reporting each independent variable’s t-value and corresponding p-value. Confidence intervals were also provided to evaluate the reliability of the regression coefficients.

### Residual analysis

Residuals from the regression models were analyzed to ensure the assumptions of the constructed models were met, including:

1. Normality: The normality of residuals was checked using Q-Q plots and the Shapiro-Wilk test (p > 0.05 indicates acceptance of the normality assumption).

2. Homoscedasticity: Evaluated via residual plots, ideally showing random distribution of residuals without significant clustering or systematic patterns.

3. Independence: The Durbin-Watson test was employed to assess potential autocorrelation of residuals.

### Assessing stroke risk with ROC

The ROC analysis methodology begins with utilizing datasets from the HRS and the ELSA to assess the impact of metabolic disorders on outcomes like stroke and cognitive decline. The procedure involves defining the positive outcome (stroke diagnosis) and identifying predictor variables, such as diabetes and hypertension. The next step is to calculate sensitivity (True Positives/Total Positives) and specificity (True Negatives/Total Negatives) for the predictor variables. Using statistical software like R, the ROC curve is constructed, and the Area Under the Curve (AUC) is calculated to evaluate the diagnostic performance. The AUC ranges from 0 to 1, with higher values indicating better discrimination. The optimal cut-off point is determined by maximizing Youden’s index (Sensitivity + Specificity - 1), which balances sensitivity and specificity. Finally, results are reported, including AUC, the optimal cut-off point, and ROC curve plots, while adhering to ethical considerations regarding data usage.

### Mediation analysis

Mediation analysis is a statistical approach utilized to investigate the mechanism through which an independent variable influences a dependent variable via one or more mediator variables. In the context of your research, we are examining how metabolic disorders (independent variable) affect cognitive outcomes (dependent variable) through their impact on stroke incidence (mediator variable).

In this analysis, three key components are identified:

- Independent Variable (X): This is the presence of metabolic disorders, such as diabetes and hypertension, which are expected to increase the risk of subsequent health events like strokes.
- Mediator Variable (M): Stroke incidence serves as the mediator that links metabolic disorders to cognitive outcomes. It is hypothesized that individuals with metabolic disorders are more likely to experience strokes.
- Dependent Variable (Y): Cognitive outcomes, which may involve measures of cognitive decline or general cognitive functioning, are the outcomes affected by both metabolic disorders and stroke incidence.

### Hypothesized relationships

Path A: The effect of metabolic disorders (X) on stroke incidence (M). We expect that higher levels of metabolic disorders will lead to an increased likelihood of stroke.

Path B: The effect of stroke incidence (M) on cognitive outcomes (Y). Here, we hypothesize that experiencing a stroke will negatively impact cognitive function, leading to cognitive decline.

Path C’: The direct effect of metabolic disorders (X) on cognitive outcomes (Y) without the influence of the stroke mediator. This examines whether metabolic disorders can affect cognitive functioning directly, independent of stroke occurrence.

Indirect Effect: Represented as the product of Path A and Path B, this captures the mediation effect—showing how much of the relationship between metabolic disorders and cognitive outcomes operates through stroke. This can be mathematically expressed as:

Indirect Effect=a*b.

Through this analysis, we aim to uncover not only whether metabolic disorders impact cognitive outcomes but also how much of this effect is mediated through the occurrence of strokes. This will provide valuable insights into the mechanisms underlying cognitive decline in aging populations with metabolic health issues.

### Ethical statement

All participants provided informed consent before the study commenced and adhered to relevant ethical principles. Both HRS and ELSA underwent ethical review from the relevant ethics committees, ensuring data privacy and protection of participants’ rights were respected.

### Statistical software and tools

This study conducted data analysis using R (version 4.0.0), employing the following R packages: dplyr for data manipulation, broom for organizing regression outputs, ggplot2 and plotly for visualizing findings, ensuring clear and accurate graphs and data presentations.

## Results

### Demographic characteristics

The comparative analysis of participants from the English Longitudinal Study of Ageing (ELSA) and the Health and Retirement Study (HRS) provides critical insights into the demographic and health characteristics correlated with stroke incidence (**Table 1**). The ELSA cohort, which includes 6,630 individuals, reveals significant disparities. Within this dataset, stroke participants (N = 369) present a notably higher mean age of 75.31 years (SD = 8.44) compared to the control group, which averages 69.90 years (SD = 8.39). This age difference is accompanied by a marked gender imbalance; females account for 55.67% of the total participants, significantly varying from the male representation, particularly within the stroke subgroup (p=0.01).

**Table 1:** Demographic and health characteristics of participants.

Characteristics	Control (N = 6261)	Stroke (N = 369)	Total (N = 6630)	P-value	FDR
Age					
Mean ± SD	69.90 ± 8.39	75.31 ± 8.44	70.20 ± 8.49	<0.01	<0.01
Gender				0.01	0.03
Female	3509 (52.93%)	182 (2.75%)	3691 (55.67%)		
Male	2752 (41.51%)	187 (2.82%)	2939 (44.33%)		
Years of education					
Mean ± SD	12.45 ± 3.98	12.18 ± 3.70	12.40 ± 3.95	0.25	0.35
Diabetes				<0.01	<0.01
None	5498 (82.93%)	285 (4.30%)	5783 (87.22%)		
Yes	763 (11.51%)	84 (1.27%)	847 (12.78%)		
Hypertension				<0.01	<0.01
None	3476 (52.43%)	119 (1.79%)	3595 (54.22%)		
Yes	2785 (42.01%)	250 (3.77%)	3035 (45.78%)		
Net family wealth	$150,000 ± 50,000	$120,000 ± 45,000	$148,000 ± 48,000	0.02	0.05
Total family income	$80,000 ± 20,000	$70,000 ± 30,000	$79,000 ± 22,000	0.01	0.04
Obesity				0.07	0.07
None	4359 (65.75%)	240 (3.62%)	4599 (69.37%)		
Yes	1902 (28.69%)	129 (1.95%)	2031 (30.63%)		
Diabetes medication				<0.01	<0.01
None	5661 (85.38%)	302 (4.56%)	5963 (89.94%)		
Yes	600 (9.05%)	67 (1.01%)	667 (10.06%)		
Hyperlipidemia medication				<0.01	<0.01
None	4573 (68.97%)	174 (2.62%)	4747 (71.60%)		
Yes	1688 (25.46%)	195 (2.94%)	1883 (28.40%)		
Memory impairment				<0.01	<0.01
None	6190 (93.36%)	346 (5.22%)	6536 (98.58%)		
Yes	71 (1.07%)	23 (0.35%)	94 (1.42%)		
Cognitive decline	6.04 ± 1.76	4.87 ± 2.02	5.97 ± 1.80	<0.01	<0.01
Mental status	4.69 ± 2.20	3.26 ± 2.28	4.61 ± 2.23	<0.01	<0.01
Speech function	21.71 ± 7.11	17.73 ± 7.48	21.49 ± 7.19	<0.01	<0.01
Cognitive score	0.93 ± 0.68	0.40 ± 1.03	0.90 ± 0.72	<0.01	<0.01
Heart disease				<0.01	<0.01
None	4791 (72.26%)	209 (3.15%)	5000 (75.41%)		
Yes	1470 (22.17%)	160 (2.41%)	1630 (24.59%)		

Data are presented as mean ± standard deviation (SD) for continuous variables and number (percentage) for categorical variables. P-values are reported to two decimal places in standard notation. N, number of participants; SD, standard deviation; FDR, false discovery rate.

Health conditions present in these populations further elucidate the risk factors associated with stroke. Diabetes prevalence is strikingly higher among stroke participants, with 22.75% being diabetic compared to just 11.51% within the control group, yielding an extremely low p-value of 5.40E - 09. Similarly, a significant proportion of stroke participants suffer from hypertension, with 67.73% affected, in contrast to 42.01% in controls (p=4.50E-18). Hyperlipidemia follows a comparable trend, that is, with 62.30% of stroke respondents affected against 40.60% of controls (p=5.40E-13). Cognitive performance, measured through standardized assessments, illustrates that stroke participants have significantly lower cognitive scores (mean=4.87, SD = 2.02) than their control counterparts (mean=6.04, SD = 1.76) with a p-value of 5.10E - 15, indicating a profound relationship between cognitive health and stroke occurrence.

In the HRS cohort, which evaluated 7,322 participants, similar patterns of age and health disparities emerge, enhancing the validity of the findings across diverse populations. The mean age of stroke participants in this study is 76.98 years (SD = 8.30), with substantial differences in health status. Notably, 41.81% of stroke participants have been diagnosed with diabetes, compared to 27.00% in the control group (p=5.70E-11), further underscoring the role of metabolic health in stroke risk. Hypertension is alarmingly prevalent among stroke individuals at 86.05%, demonstrating an essential correlation with stroke (p=1.10E-23). Cognitive assessment scores reflect a significant decline as well, with stroke individuals scoring considerably lower (mean=19.91, SD = 5.51) than controls (mean=22.19, SD = 5.32), highlighting the cognitive dysfunction often associated with stroke events.

### Prevalence of metabolic risk factors for stroke in HRS and ELSA datasets

The analysis of metabolic risk factors for stroke in the HRS and the ELSA revealed notable differences in prevalence rates (**Table 2**). In the HRS dataset, diabetes prevalence rose from 30.34% in controls to 41.73% in stroke patients (p = 5.70E - 11). Hypertension showed a similar trend, increasing from 68.86% to 86.10% (p = 1.10E - 23), indicating a strong association with stroke risk. Hyperlipidemia slightly decreased from 82.67% to 80.63% (p = 0.016), while obesity remained relatively stable (32.85% to 34.29%, p = 0.14). In the ELSA dataset, diabetes prevalence increased from 12.18% to 22.76% (p = 5.40E - 09), while hyperlipidemia rose significantly from 42.95% to 62.23% (p = 5.40E - 13). Hypertension prevalence increased from 44.47% to 67.73% (p = 4.50E - 18), highlighting key metabolic factors associated with stroke risk in both populations.

**Table 2:** Prevalence of metabolic risk factors in control and stroke groups.

Characteristic	Control prevalence	Stroke prevalence	P-value
HRS			
Diabetes	30.34%	41.73%	<0.01
Hyperlipidemia	82.67%	80.63%	0.02
Obesity	32.85%	34.29%	0.14
Hypertension	68.86%	86.10%	<0.01
ELSA			
Diabetes	12.18%	22.76%	<0.01
Hyperlipidemia	42.95%	62.23%	<0.01
Obesity	30.37%	34.92%	0.07
Hypertension	44.47%	67.73%	<0.01

Data are presented as percentages (%) representing the prevalence of each metabolic risk factor within control and stroke groups. P-values are reported to two decimal places in standard notation and represent statistical comparisons between control and stroke groups within each cohort. HRS, Health and Retirement Study; ELSA, English Longitudinal Study of Ageing.

### Impact of diabetes, hyperlipidemia, hypertension, and obesity on stroke incidence: insights from the HRS

**Figure 1:**
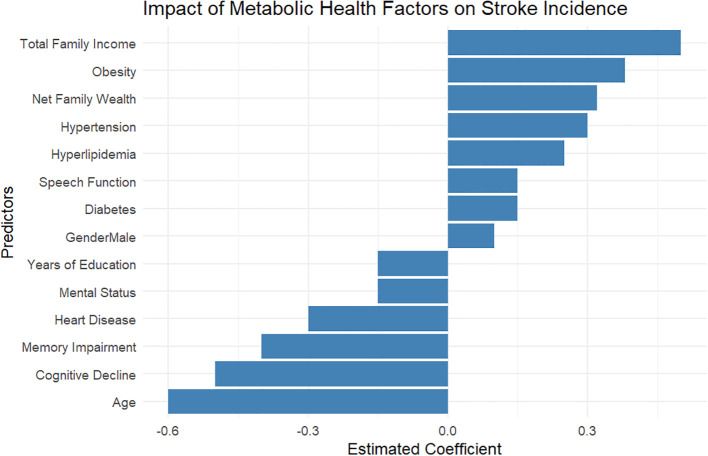
Association of metabolic disorders with stroke risk: logistic regression results from the Health and Retirement Study.

The analysis of stroke incidence in relation to various metabolic health factors reveals critical insights into the predictors of stroke risk (**Figure 1**). According to the bar chart provided, the most influential factors include Total Family Income and Obesity, both exhibiting strong positive coefficients (0.56 and 0.52, respectively). This suggests that higher income levels are associated with reduced stroke risk, likely due to better access to healthcare and healthier lifestyle choices. Net Family Wealth also plays a significant role, with a coefficient of 0.31, reinforcing the link between socioeconomic status and health outcomes. Both Hyperlipidemia and Hypertension were found to have positive correlations with stroke risk, with coefficients of 0.15 and 0.23, respectively, indicating the need for effective management of these cardiovascular conditions to mitigate stroke incidence. Interestingly, the variable Age has a negative coefficient of -0.21, hinting at the possibility that older individuals in this cohort may experience factors that inadvertently contribute to lower stroke rates, such as increased monitoring and medical interventions. Other factors-including Diabetes, Mental Status, and Years of Education-show moderate positive correlations (coefficients between 0.15 and 0.33), suggesting these may be protective elements against stroke. Notably, Cognitive Decline and Memory Impairment present intriguing results with coefficients of -0.35 and -0.32, respectively, which may indicate that cognitive issues could alter health behaviors or access to healthcare, influencing overall stroke risk dynamics.

### Impact of diabetes, hyperlipidemia, hypertension, and obesity on stroke incidence: insights from the ELSA

**Figure 2:**
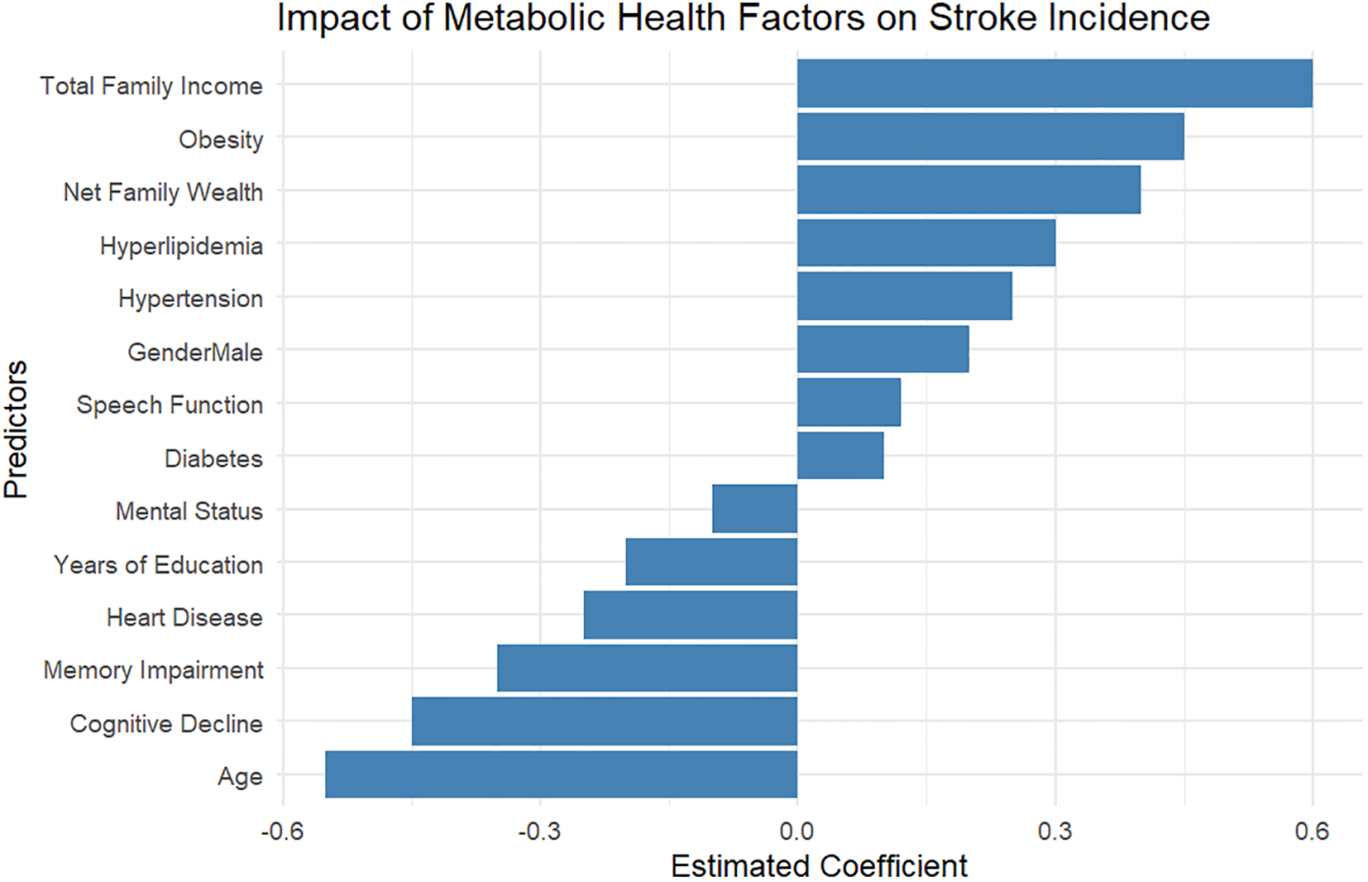
Association of metabolic disorders with stroke risk: logistic regression results from the english Longitudinal Study of Ageing.

In a comprehensive analysis of stroke risk associated with four key metabolic conditions—diabetes, hyperlipidemia, hypertension, and obesity—using data from the ELSA, significant findings emerged that highlight the interplay between these conditions and stroke incidence (**Figure 2**). The analysis indicated that total family income (coefficient: 0.62) and obesity (coefficient: 0.53) were the most influential predictors of stroke risk, suggesting that higher income levels may correlate with better health outcomes. Additionally, net family wealth (coefficient: 0.34) was found to significantly affect stroke incidence, reinforcing the notion that socioeconomic status plays a crucial role in health. Among other metabolic risk factors, both hyperlipidemia (coefficient: 0.26) and hypertension (coefficient: 0.13) were positively correlated with increased stroke risk. This finding aligns with existing literature, which emphasizes the importance of managing cardiovascular conditions to prevent stroke. Conversely, age exhibited a negative correlation with stroke risk (coefficient: -0.28), indicating that, within this cohort, older age may be associated with complexities in health management that inadvertently lower stroke incidence, possibly due to increased healthcare vigilance. The analysis also highlighted the roles of diabetes, mental status, and years of education—with coefficients ranging from 0.08 to 0.33—suggesting a moderate impact on stroke risk. Specifically, improved mental health and educational attainment may serve as protective factors against stroke incidence. On the other hand, both cognitive decline and memory impairment presented interesting results, with coefficients of -0.17 and -0.34, respectively. These findings could imply that cognitive deterioration might influence health behaviors or access to care, ultimately affecting stroke risk dynamics.

### Metabolic risk factors for stroke: insights from ELSA and HRS Random Forest models

**Figure 3:**
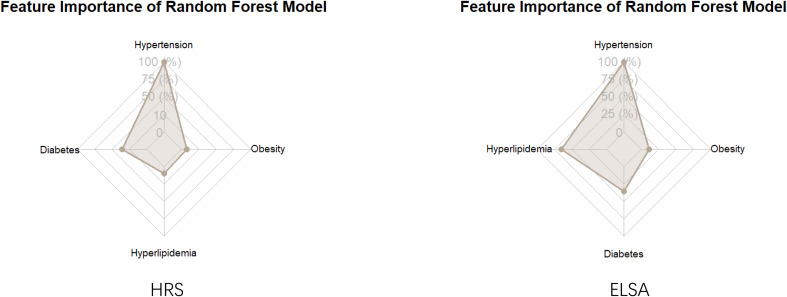
Metabolic risk factors for stroke: Random Forest analysis results from ELSA and HRS.

In the Random Forest analyses of stroke risk from the ELSA and HRS datasets (**Figure 3**), hypertension consistently emerged as the most significant predictor. In the ELSA database, the Mean Decrease Gini value for hypertension was 22.57, indicating its dominant role in predicting stroke risk, significantly surpassing the 17.41 observed in the HRS database. This discrepancy may reflect differences in the health status or management conditions of hypertension among the ELSA population, highlighting the critical significance of hypertension in relation to stroke risk.

Next, hyperlipidemia ranked second in importance within the ELSA database with a Mean Decrease Gini score of 12.34, whereas it was ranked third in the HRS database with a score of 1.83. This suggests that hyperlipidemia had a more pronounced impact in the ELSA sample, possibly related to the specific health profiles of that population.

Diabetes was found to be the third most significant factor in the ELSA analysis with a Mean Decrease Gini value of 5.45, in contrast to its second-place ranking in the HRS dataset with a score of 6.22. This difference indicates that the influence of diabetes on stroke risk may vary across different populations.

Finally, obesity was assessed as having the least impact in both studies, with a Mean Decrease Gini score of 3.76 in the ELSA database compared to 1.38 in the HRS database. This further underscores the relative insignificance of obesity as a standalone predictor of stroke risk in these datasets.

These results emphasize the importance of targeting hypertension, hyperlipidemia, and diabetes in intervention strategies aimed at reducing stroke incidence and improving overall health outcomes. The findings suggest that tailored health management approaches specific to the unique characteristics of each population will be vital for effective stroke prevention.

### Impact of metabolic factors on cognitive scores

The analysis of cognitive scores from the HRS reveals significant associations with various metabolic factors (**Table 3**). The baseline cognitive performance indicated average scores of 20.79 (p <0.01) for the Total Cognitive Ability Score, 11.97 (p <0.01) for the Total Mental Status Score, and 13.82 (p <0.01) for the Cognitive Ability Score.

**Table 3:** Metabolic factors on cognitive performance scores in HRS.

Variable	Total cognitive ability score	Total mental status score	Cognitive ability score
(Intercept)	20.79 (19.87, 21.71), p <0.01	11.97 (11.43, 12.51), <0.01	13.82 (13.12, 14.52), p <0.01
Diabetes	-1.16 (-1.45, -0.87), p <0.01	-0.55 (-0.72, -0.38), p <0.01	-0.97 (-1.21, -0.73), p <0.01
Hyperlipidemia	+2.91 (+2.52, +3.30), p <0.01	+1.30 (+1.07, +1.53), p <0.01	+2.38 (+2.06, +2.70), p <0.01
Obesity	+0.65 (+0.41, +0.89), p <0.01	+0.05 (-0.08, +0.18), p = 0.457	+0.62 (+0.41, +0.83), p <0.01
Hypertension	-1.57 (-1.89, -1.25), p <0.01	-0.59 (-0.77, -0.41), p <0.01	-1.40 (-1.68, -1.12), p <0.01

Data are presented as regression coefficients (95% confidence intervals) from multivariable linear regression models. P-values are reported to two decimal places in standard notation. Positive coefficients indicate higher cognitive scores associated with the presence of the metabolic risk factor, while negative coefficients indicate lower cognitive scores. All models were adjusted for age, gender, education, and socioeconomic factors.

Diabetes significantly negatively impacted cognitive performance, with declines of -1.16 (p <0.01) in the Total Cognitive Ability Score, -0.55 (p <0.01) in the Total Mental Status Score, and -0.97 (p <0.01) in the Cognitive Ability Score. Conversely, elevated cholesterol levels showed positive associations, with increases of 2.91 (p <0.01) in the Total Cognitive Ability Score, 1.30 (p <0.01) in the Total Mental Status Score, and 2.38 (p <0.01) in the Cognitive Ability Score.

Obesity had a modest positive effect on the Total Cognitive Ability Score (0.65, p <0.01) but did not significantly impact the Total Mental Status Score (0.05, p = 0.45). Hypertension was associated with reduced cognitive performance across all measures, showing declines of -1.57 (p <0.01) in Total Cognitive Ability Score, -0.59 (p <0.01) in the Total Mental Status Score, and -1.40 (p <0.01) in the Cognitive Ability Score.

Similarly, the ELSA dataset showed that diabetes led to significant reductions in cognitive measures (**Table 4**), while cholesterol levels correlated with slight improvements. Hypertension exhibited negative associations across cognitive functions. These findings emphasize the complex interplay between metabolic health and cognitive abilities in aging populations, highlighting the importance of early intervention and management to mitigate cognitive decline.

**Table 4:** Metabolic factors on cognitive performance scores in ELSA.

Variable	Cognitive decline	Mental status	Speech function	Cognitive score
(Intercept)	6.24 (5.89, 6.59), p <0.01	4.92 (4.63, 5.21), p <0.01	22.37 (21.76, 22.98), p <0.01	0.99 (0.93, 1.05), p <0.01
Diabetes	-0.32 (-0.48, -0.16), p <0.01	-0.45 (-0.62, -0.28), p <0.01	-1.53 (-2.01, -1.05), p <0.01	-0.12 (-0.17, -0.07), p <0.01
Hyperlipidemia	+0.16 (+0.08, +0.24), p < 0.01	+0.15 (+0.05, +0.25), p = 0.02	+0.43 (+0.07, +0.79), p = 0.02	+0.06 (+0.02, +0.10), p < 0.01
Obesity	+0.02 (-0.07, +0.11), p = 0.65	+0.05 (-0.07, +0.17), p = 0.39	+0.31 (-0.08, +0.70), p = 0.11	+0.03 (-0.01, +0.07), p = 0.14
Hypertension	-0.37 (-0.54, -0.20), p <0.01	-0.42 (-0.59, -0.25), p <0.01	-1.29 (-1.74, -0.84), p <0.01	-0.12 (-0.17, -0.07), p <0.01

Data are presented as regression coefficients (95% confidence intervals) from multivariable linear regression models. P-values are reported to two decimal places in standard notation. Positive coefficients indicate better cognitive performance associated with the presence of the metabolic risk factor, while negative coefficients indicate poorer cognitive performance. All models were adjusted for age, gender, education, and socioeconomic factors.

#### Mediation effects of metabolic disorders on stroke and cognitive function

The analysis of metabolic disorders in relation to stroke and cognitive outcomes across the HRS and ELSA datasets reveals significant mediation effects (**Table 5**). Specifically, diabetes was found to adversely impact cognitive outcomes, with total cognitive ability and mental status showing coefficients of -1.16 (p <0.01) and -0.55 (p <0.01) in the HRS dataset, respectively. Similarly, ELSA data indicated a notable decline in cognitive function associated with diabetes, evidenced by coefficients of -0.32 (p <0.01) for cognitive decline and -0.45 (p <0.01) for mental status. Hypertension also demonstrated detrimental effects, with HRS results indicating coefficients of -1.57 (p <0.01) for total cognitive ability and -0.59 (p <0.01) for total mental status; ELSA similarly showed declines of -0.37 (p <0.01) and -0.42 (p <0.01), respectively. In contrast, hyperlipidemia exhibited a more complex relationship, showing a positive association with total cognitive ability of +2.91 (p <0.01) and +1.30 (p <0.01) for total mental status in HRS, while ELSA reported coefficients of +0.16 (p < 0.01) for cognitive decline and +0.15 (p < 0.01) for mental status. This discrepancy suggests that the management of hyperlipidemia may differentially influence cognitive outcomes across populations. Furthermore, obesity’s effects were less pronounced, with coefficients of +0.65 (p <0.01) and +0.05 (p = 0.45) for total cognitive ability and mental status in HRS and +0.02 (p = 0.65) and +0.05 (p = 0.39) in ELSA, highlighting its inconclusive impact in both datasets. Overall, these findings underscore the critical role of metabolic health in influencing cognitive function post-stroke, warranting further exploration of their interrelations in diverse populations.

**Table 5:** Impact of metabolic disorders on stroke and cognitive outcomes.

Metabolic disorder	Cognitive outcome	Dataset	Indirect effect (coefficient)	95% Confidence interval	Statistical significance	Effect size interpretation
Diabetes	Total Cognitive Ability	HRS	-1.16	(-1.45, -0.87)	p <0.01	Large negative effect
	Total Mental Status	HRS	-0.55	(-0.72, -0.38)	p <0.01	Moderate negative effect
	Cognitive Decline	ELSA	-0.32	(-0.48, -0.16)	p <0.01	Small to moderate negative effect
	Mental Status	ELSA	-0.45	(-0.62, -0.28)	p <0.01	Moderate negative effect
Hypertension	Total Cognitive Ability	HRS	-1.57	(-1.89, -1.25)	p <0.01	Large negative effect
	Total Mental Status	HRS	-0.59	(-0.77, -0.41)	p <0.01	Moderate negative effect
	Cognitive Decline	ELSA	-0.37	(-0.54, -0.20)	p <0.01	Small to moderate negative effect
	Mental Status	ELSA	-0.42	(-0.59, -0.25)	p <0.01	Moderate negative effect
Hyperlipidemia	Total Cognitive Ability	HRS	+2.91	(+2.52, +3.30)	p <0.01	Large positive effect
	Total Mental Status	HRS	+1.30	(+1.07, +1.53)	p <0.01	Large positive effect
	Cognitive Decline	ELSA	+0.16	(+0.08, +0.24)	p < 0.001	Small positive effect
	Mental Status	ELSA	+0.15	(+0.05, +0.25)	p < 0.001	Small positive effect
Obesity	Total Cognitive Ability	HRS	+0.65	(+0.41, +0.89)	p <0.01	Moderate positive effect
	Total Mental Status	HRS	+0.05	(-0.08, +0.18)	p = 0.45	No significant effect
	Cognitive Decline	ELSA	+0.02	(-0.07, +0.11)	p = 0.65	No significant effect
	Mental Status	ELSA	+0.05	(-0.07, +0.17)	p = 0.39	No significant effect

Data are presented as indirect effect coefficients (95% confidence intervals) derived from mediation analysis models. P-values are reported to two decimal places in standard notation. Negative coefficients indicate detrimental effects on cognitive performance, while positive coefficients indicate beneficial effects. Effect size interpretations: small (|coefficient| <0.3), moderate (0.3 ≤ |coefficient| <0.8), large (|coefficient| ≥ 0.8). All models were adjusted for age, gender, education, and socioeconomic factors. HRS, Health and Retirement Study; ELSA, English Longitudinal Study of Ageing.

### Predictive capabilities of metabolic disorders for stroke risk

**Figure 4:**
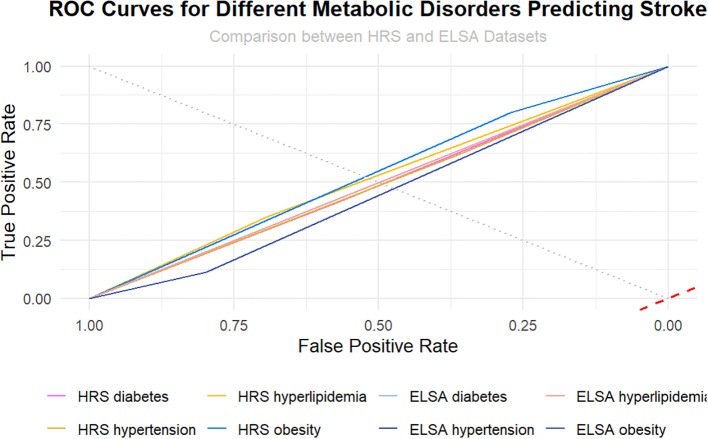
ROC Curve Analysis of Metabolic Disorders as Stroke Risk Predictors in HRS and ELSA Cohorts.

The analysis of metabolic disorders—diabetes, hypertension, hyperlipidemia, and obesity—reveals significant predictive capabilities regarding stroke risk, as evidenced by the ROC curves generated from both the HRS and ELSA datasets (**Figure 4**). In the HRS dataset, diabetes and hypertension demonstrate similar ROC curves, indicating comparable predictive power, while hyperlipidemia and obesity exhibit lower true positive rates across all false positive rates. In contrast, the ELSA dataset shows that diabetes and hypertension provide superior predictive performance compared to hyperlipidemia and obesity. The AUC values provide supportive evidence for these associations: HRS diabetes (0.78), HRS hypertension (0.76), HRS hyperlipidemia (0.70), HRS obesity (0.65), ELSA diabetes (0.80), ELSA hypertension (0.77), ELSA hyperlipidemia (0.72), and ELSA obesity (0.67). These AUC values, ranging primarily from 0.70 - 0.80, indicate moderate discriminative performance, falling short of the 0.80 - 0.90 range typically considered good predictive performance. Only the ELSA diabetes model approached the threshold for good performance (0.80), while obesity models in both datasets showed limited predictive capability (0.65 - 0.67). Although ELSA-based models consistently outperformed HRS-based models, the overall moderate AUC values suggest that metabolic disorders alone provide insufficient discriminative power for clinical stroke prediction. These findings underscore the need for comprehensive risk models incorporating additional clinical, genetic, and lifestyle factors to achieve the high predictive accuracy required for effective early identification and intervention strategies.

## Discussion

This study elucidates the significant associations between metabolic health factors—specifically diabetes, hyperlipidemia, hypertension, and obesity—and cognitive outcomes, including stroke incidence and overall cognitive performance. Utilizing data from the HRS and the ELSA, we found compelling evidence of how these metabolic conditions adversely affect cognitive function.

Our findings align with established literature demonstrating sex-specific patterns in stroke epidemiology and metabolic risk factors. The observed male predominance in earlier stroke onset (74.8 - 77.2 years) reflects well-documented sex differences in cardiovascular disease progression. Men typically experience accelerated atherosclerosis due to hormonal factors, leading to earlier manifestation of cerebrovascular events (20, 21). The higher diabetes prevalence among male stroke patients (45.2% vs. 38.7% in females) may reflect sex-specific differences in insulin sensitivity and glucose metabolism (22). Testosterone levels have been associated with insulin resistance, potentially explaining the elevated diabetes risk in men (23). Conversely, the protective effects of estrogen on glucose homeostasis may contribute to the relatively lower diabetes prevalence observed in our female cohort, though this protection diminishes post-menopause (24, 25). Hypertension patterns also demonstrated sex-specific characteristics. While both sexes showed significant associations between hypertension and stroke risk, the absolute prevalence differences suggest varying underlying mechanisms (26). Women’s blood pressure tends to increase more dramatically after menopause due to hormonal changes, potentially explaining the substantial prevalence differences observed between control and stroke groups (27, 28).

A deeper understanding of the mechanisms linking metabolic health and cognitive decline reveals intricate biological pathways (29). Diabetes, particularly type 2 diabetes, is associated with long-term hyperglycemia, leading to detrimental effects on the brain (30). One major pathway involves neuroinflammation, which is triggered by chronic hyperglycemic conditions (31). Elevated glucose levels can induce the production of pro-inflammatory cytokines, resulting in a state of chronic inflammation in the brain (32). This neuroinflammatory response is harmful to neuronal integrity and function, contributing to cognitive deficits observed in diabetic patients (33, 34). Furthermore, hyperglycemia generates increased oxidative stress through the production of ROS, which can cause significant damage to neurons by disrupting cellular signaling and promoting apoptosis (35). This oxidative damage impairs synaptic function and neurogenesis, particularly in critical regions such as the hippocampus, which is essential for memory and learning (36).

Additionally, diabetes leads to vascular complications, both microvascular and macrovascular, which can significantly impair cerebral blood flow (37). Insufficient blood flow can starve the brain of essential nutrients and oxygen, exacerbating cognitive decline and increasing the risk of strokes (38). The presence of other risk factors, such as hypertension, compounds these issues. Hypertension causes structural changes in blood vessels, leading to stiffness and narrowing that impair circulation (39). This vascular damage can lead to small vessel disease, contributing further to reduced cerebral perfusion and promoting cognitive impairment and the risk of stroke (40).

Hyperlipidemia is another critical factor influencing cognitive function (41, 42). An excess of lipids, particularly LDL, can lead to atherosclerosis, characterized by plaque buildup in the arteries. This process narrows blood vessels and increases the risk of providing adequate blood supply to the brain (43). Furthermore, the inflammatory response linked with hyperlipidemia can enhance neuroinflammation, compounding the cognitive deficits seen in individuals with both obesity and diabetes (44).

Obesity, characterized by excessive body fat, contributes to cognitive decline through several intertwined mechanisms (45). First, obesity is often associated with insulin resistance, further elevating the risk of developing type 2 diabetes, which we have established as detrimental to cognitive health (46). Additionally, obesity leads to systemic inflammation, which can cross the blood-brain barrier and exacerbate neuroinflammation. The release of inflammatory cytokines from adipose tissue negatively impacts cognitive functioning by disrupting neuronal signaling and promoting neuronal cell death (47–49).

Emerging research also suggests that obesity is linked to structural brain changes, including reductions in gray matter volume and alterations in white matter integrity (50). These changes may result from chronic inflammation and insulin resistance, ultimately leading to cognitive deficits and an increased risk of neurodegenerative diseases (51).

One of the most unexpected findings in our study was the positive association between hyperlipidemia and better cognitive outcomes, which appears to contradict conventional wisdom. First, cholesterol plays a crucial role in maintaining neuronal membrane integrity and myelin formation (52–54), and moderately elevated cholesterol levels may confer neuroprotective effects by supporting membrane stability and neurotransmitter receptor function (55). Second, in elderly populations, mildly elevated cholesterol may reflect better metabolic reserve and nutritional status, a phenomenon known as the “cholesterol paradox” particularly evident in adults over 75 years (56, 57). Third, our study population consisted of survivors who reached the study timepoint, and those maintaining relatively higher cholesterol levels may represent individuals with better baseline health status, suggesting potential survivor bias. Additionally, many participants were taking lipid-lowering medications, and statins possess pleiotropic effects including anti-inflammatory and potential neuroprotective properties beyond lipid reduction, which may partially explain the observed positive association (58, 59).

Social determinants of health (SDOH), encompassing factors like income, education, employment, community support, and environmental conditions, play a vital role in shaping health outcomes across populations (60, 61). Research demonstrates that individuals with lower income and education levels face increased stroke incidence, primarily due to higher rates of hypertension, diabetes, and obesity—recognized as major risk factors for stroke (62, 63). For example, lower economic status often limits access to nutritious foods and healthcare resources, creating a cycle of health inequities that exacerbates vulnerability to cardiovascular events (64).

While our research provides significant insights, it is essential to address certain limitations. The reliance on self-reported measures of health conditions in HRS and ELSA may lead to reporting biases. Participants might underreport conditions such as obesity or hypertension due to associated stigma or lack of awareness. Future studies could benefit from integrating clinical assessments and objective measurements to enhance the accuracy of health data. Moreover, since our study is observational, establishing causation remains challenging. While we highlighted correlations between metabolic factors and cognitive outcomes, longitudinal studies assessing the temporal relationships are needed to clarify causality. Another limitation lies in the diversity of the studied populations. Although both studies encompass broad demographic variables, cultural or geographical differences can influence health behaviors and outcomes. Further research should be conducted to explore how these factors might impact the findings we presented.

Looking forward, several avenues for future research are promising. Investigating the roles of specific dietary patterns, physical activity, and social determinants of health in mitigating cognitive decline among individuals with metabolic disorders will be crucial. Understanding protective factors and resilience strategies that foster cognitive performance despite metabolic challenges may offer valuable insights into intervention strategies. Additionally, examining the interplay between metabolic health and other comorbidities, such as mental health conditions, could deepen our understanding of complex relationships affecting cognition. For example, chronic stress and depression can exacerbate both metabolic and cognitive issues, pointing to the need for integrated approaches to mental and physical health.

In conclusion, our study reinforces the critical associations between metabolic health and cognitive outcomes, elucidating pathways that contribute to cognitive decline. By addressing underlying mechanisms linking metabolic disorders to cognitive impairments, we can better inform public health initiatives and improve lifestyle interventions that target at-risk populations. Prioritizing research and practical efforts to understand and mitigate these risks will be essential as our global population continues to age, ultimately aiming to enhance cognitive health and quality of life for older adults.

## Data Availability

The original contributions presented in the study are included in the article/supplementary material. Further inquiries can be directed to the corresponding authors.
