# Correction: The interplay between metabolic health factors and stroke incidence in aging populations

**DOI:** 10.3389/fendo.2025.1699622

**Published:** 2025-12-19

**Authors:** Chonghui Zhang, Tao Xiong, Kaili Ren, Hongyu Wu, Shanshan Cai, Liqin Wang

**Affiliations:** 1 Department of Blood Transfusion, The Affiliated Hospital of Qingdao University, Qingdao, China; 2 Nanjing Medical University, Nanjing, China; 3 Division of Biomedical and Life Sciences, Faculty of Health and Medicine, Lancaster University, Lancaster, United Kingdom

**Keywords:** Keywords: metabolic disorders, stroke risk, diabetes, hypertension, obesity

The figures were in the wrong order in all versions of this paper. The correct order appears below. The citations of the figures will not be updated as these were correct.

The original article has been updated.

## Figures and Tables

**Figure 1:**
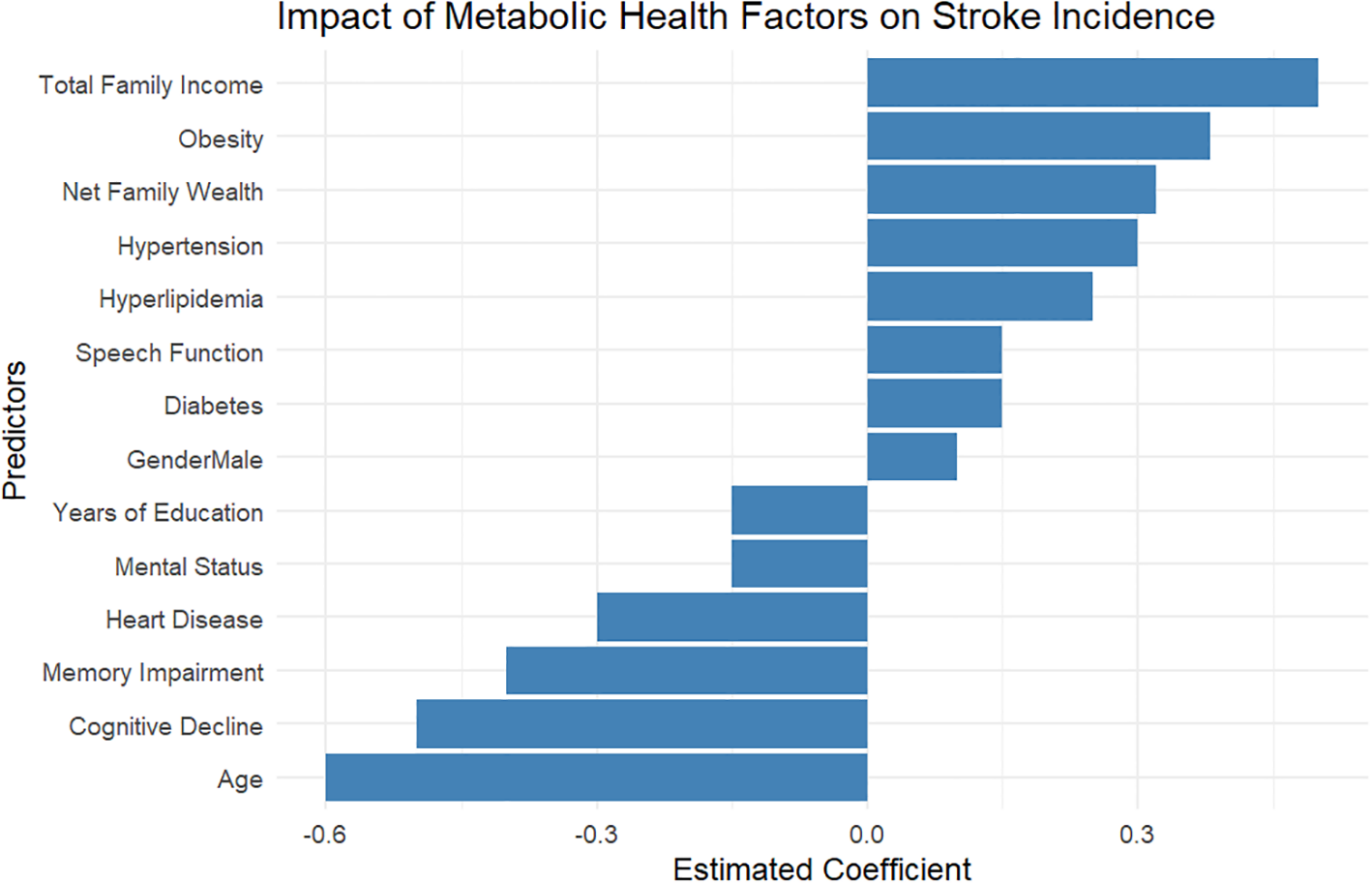
Association of metabolic disorders with stroke risk: logistic regression results from the Health and Retirement Study.

**Figure 2:**
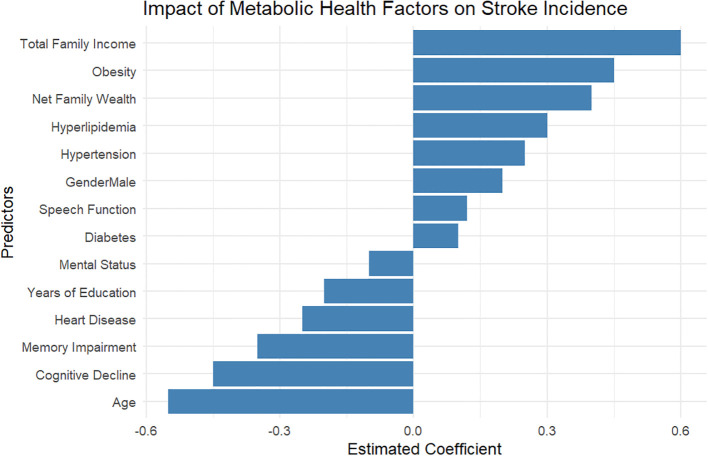
Association of metabolic disorders with stroke risk: logistic regression results from the english Longitudinal Study of Ageing.

**Figure 3:**
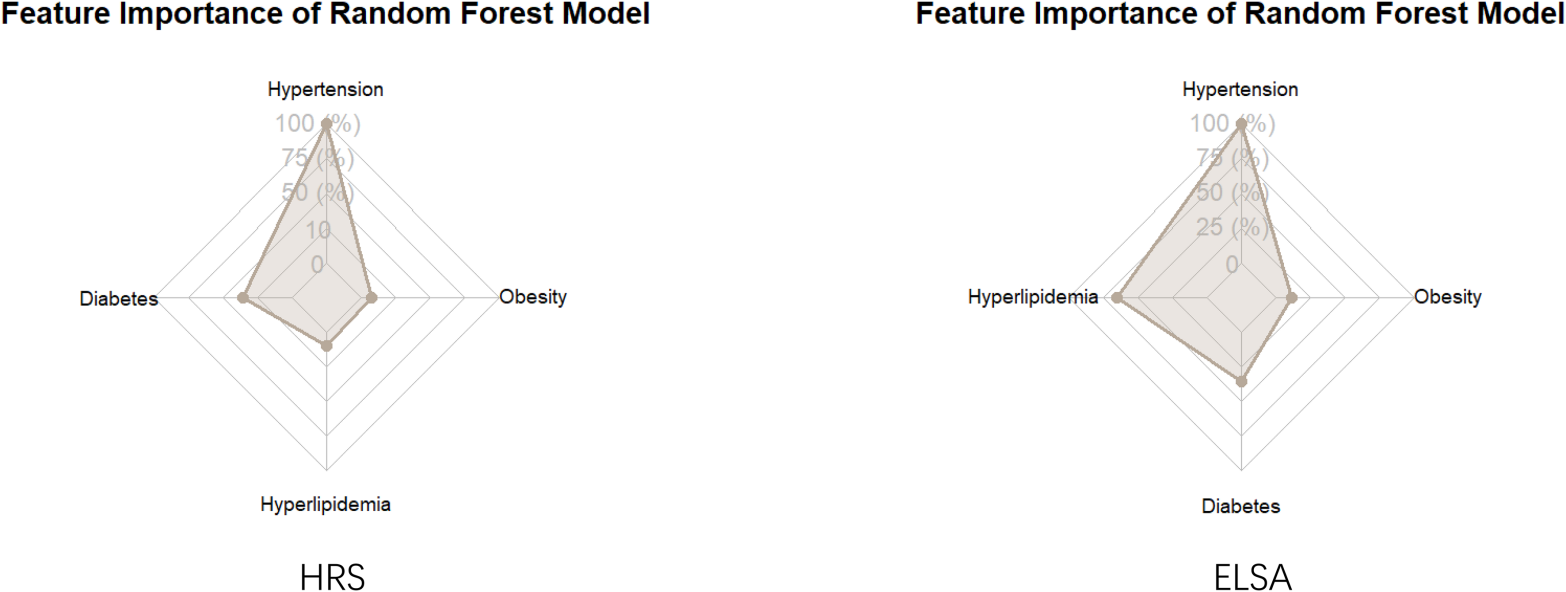
Metabolic risk factors for stroke: Random Forest analysis results from ELSA and HRS.

**Figure 4:**
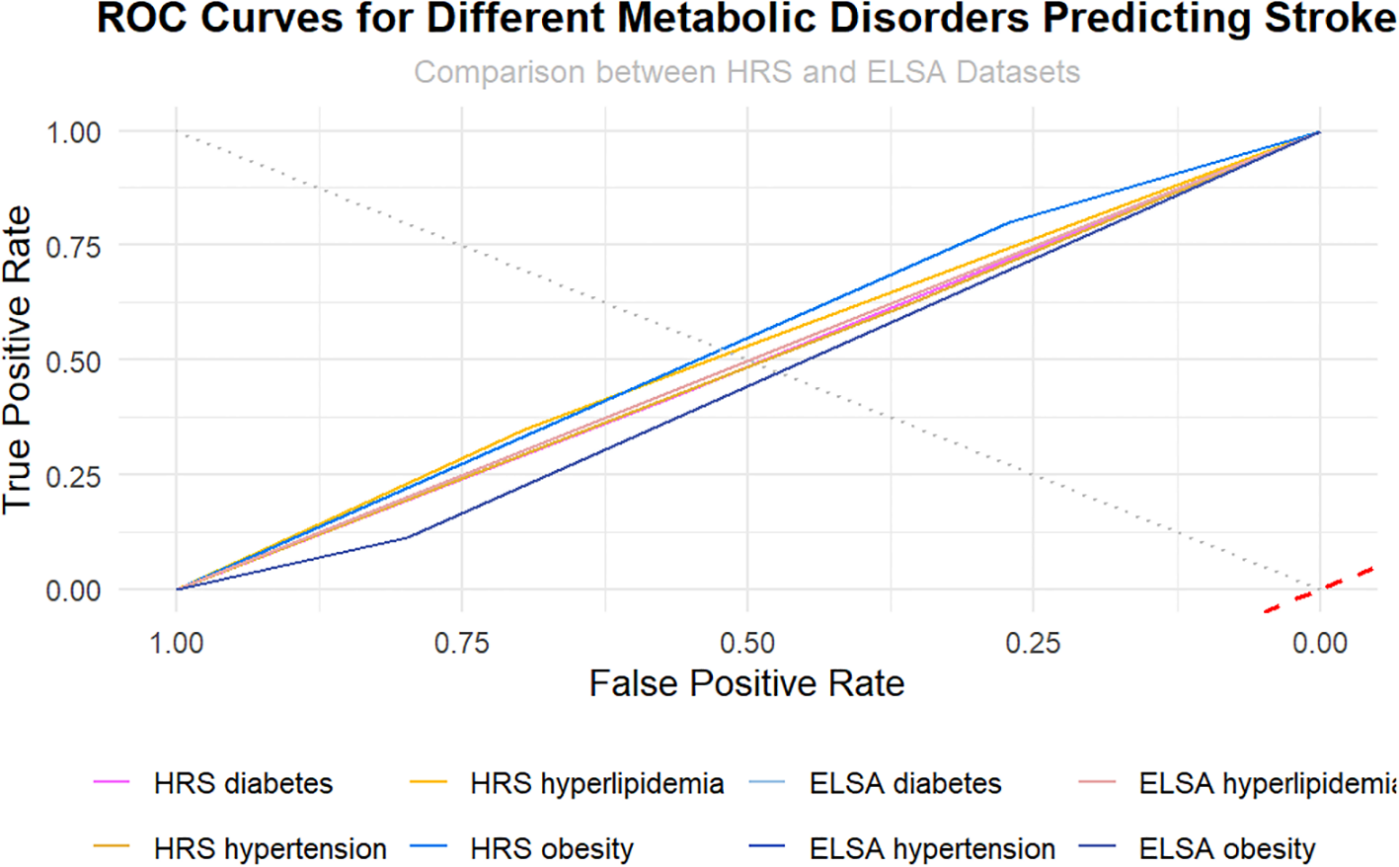
ROC Curve analysis of metabolic disorders as stroke risk predictors in HRS and ELSA cohorts.

